# Sassy: fuzzy searching DNA sequences using SIMD

**DOI:** 10.1093/bioinformatics/btag244

**Published:** 2026-05-24

**Authors:** Rick Beeloo, Ragnar Groot Koerkamp

**Affiliations:** Department of Theoretical Biology and Bioinformatics, Utrecht University, Utrecht, 3584CH, Netherlands; Department of Computer Science, ETH Zurich, Zurich, 8092, Switzerland; Department of Computer Science, Karlsruhe Institute of Technology, Karlsruhe, 76131, Germany

## Abstract

**Motivation:**

Approximate string matching (ASM) is the problem of finding all occurrences of a pattern in a text while allowing up to *k* errors. Many modern methods use *seed-chain-extend*, which is fast in practice, but does not guarantee finding *all* matches with ≤k errors. However, applications such as CRISPR off-target detection require exhaustive results.

**Results:**

We introduce Sassy, a library and tool for ASM of short patterns in long texts. Sassy splits the text into four parts that are searched in parallel, and uses bitvectors in the text direction rather than the pattern direction. This has complexity O(k⌈n/W⌉) when searching a random text of length *n*, where W=256 is the SIMD width, and provides significant speedups for small *k*. Separately, we allow matches of the pattern to extend beyond the text for an *overhang cost* of, e.g. α=0.5 per character, to find matches near contig or read ends.

Sassy is 4× to 15× faster than Edlib for patterns ≤1000 bp, and can search text with a throughput near 2 Gbp/s. Likewise, Sassy is over 100× faster than parasail. We apply Sassy to CRISPR off-target detection by searching 61 guide sequences in a human genome. Sassy is 100× faster than SWOffinder and only slightly slower (for k≤3) than CHOPOFF, for which building its index takes 20 min. Sassy also scales well to larger *k*, unlike CHOPOFF whose index took over 10 h to build for k=5.

**Availability and implementation:**

Sassy is available as library and binary at https://github.com/RagnarGrootKoerkamp/sassy, and archived at swh:1:dir:e884758dce5777a441bc2799dc8824e563c5f97b.

## 1 Introduction

Approximate string matching (ASM) is the problem of finding all matches of a pattern *P* of length *m* in a text *T* of length *n* with at most *k* errors (Navarro 2001). In this article, we consider errors under the unit-cost edit distance, also known as Levenshtein distance (Levenshtein *et al.* 1966). ASM has applications in many different fields. Specifically in bioinformatics, instances of ASM are CRISPR off-target detection (Yaish *et al.* 2024, Roux *et al.* 2025) and searching barcodes for demultiplexing (Cheng *et al.* 2024, Beeloo *et al.* 2025).

Recent years have seen a large number of papers on speeding up the related problem of (semi-)global alignment by using faster implementations (bitpacking, SIMD), faster algorithms (A*), and better banding heuristics (see Section 1.2). Simultaneously, there is a lot of research on *mapping*: aligning, say, 1 kbp reads against static text indices that can range from megabases to gigabases in size, without the guarantee of finding *all* matches. Since this guarantee is important for many bioinformatic applications, we identify that there is no modern, SIMD-based tool for ASM. Sassy (SIMD Approximate String Searcher) fills this gap.

### 1.1 Contributions

Sassy is a conceptually simple but highly efficient command line tool and Rust library for ASM. Sassy targets patterns with length up to around a 2000 characters. It supports both ASCII and (IUPAC) DNA sequences, runs on both AVX2 (x86-64) and NEON (ARM), and comes with C and Python bindings. The underlying algorithm does not require a precomputed text index and instead can operate directly on the records of an input stream. This makes it especially suitable for, e.g. searching a pattern while streaming DNA reads, one-off searches in assembled genomes, and reference-free analysis.

On a high level, our main contributions are:

1. We argue that while similar, semi-global alignment, mapping, and ASM are all distinct problems, and that for certain applications, exact methods for ASM are required and currently not available.
2. We define what it means to “report all matches”, and choose to report only local minima by default. We note that reverse-complementing inputs can give different results (Fig. 2).
3. We optimize ASM for searching through long texts. Algorithmically this has two small novelties: (i) bitpacking in the text direction, rather than the pattern direction, and (ii) intra-sequence parallelism by splitting the text into four chunks that are processed in parallel using W=256-bit SIMD. This leads to expected-case complexity O(k⌈n/W⌉) when matching against random text, and O(m⌈n/W⌉) in the worst-case when excluding the time for tracebacks.
4. In Appendix B, available as supplementary data at *Bioinformatics* online, we introduce an *overhang cost* α=0.5 that allows and controls the cost of *overhanging* alignments extending beyond the text.
5. Sassy is 4−15× faster than Edlib for patterns up to length 1000 with up to 5% divergence.
6. Sassy is 100× faster than Swoffinder for CRISPR off-target detection, and equally fast or faster than the index-based CHOPOFF while reporting identical matches.

### 1.2 Previous work

ASM has been extensively studied between 1980 and 2000, mostly concluding in the bitpacking algorithm of Myers (1999). For word size w=64 (compared to SIMD ′width W=256), this has worst-case complexity O(⌈m/w⌉n), or expected-case complexity O(⌈k/w⌉n) on random text. Since then, research has shifted to other types of pairwise alignment. Indeed, both *global alignment* and *mapping* are very active areas of research on similar but slightly different problems. Some methods developed for those problems can also be applied to ASM. Unfortunately, they usually do not guarantee to return all matches, either because they only return best matches, as in *semi-global alignment*, or because of their heuristic nature in case of mappers. Before discussing the older results on ASM itself in detail, we first cover some recent work on these related problems, so that the differences can be appreciated.

#### 1.2.1 Global alignment

In *global alignment*, the pattern *P* is aligned against the *entire* text *T*. The lengths m:=|P| and n:=|T| are typically relatively close to each other, and may range from tens to millions of bases. The classical Needleman–Wunsch (Needleman and Wunsch 1970) or Wagner–Fischer/Levenshtein (Wagner and Fischer 1974, Levenshtein *et al.* 1966) algorithm requires O(nm) time and space, although space can be reduced to O(min(m,n)) when only the alignment cost is needed. While no algorithm breaks the worst-case $O(n^{2-\varepsilon })$ barrier under SETH (Backurs and Indyk 2015), many practical methods achieve sub-quadratic performance on typical inputs. For instance, Ukkonen’s band-doubling method (Ukkonen 1985b) runs in O(ns) time, where *s* is the edit distance, and diagonal-transition approaches (Myers 1986, Ukkonen 1985a) attain $O(n+s^{2})$ both in expectation on random texts and in practice. A recent implementation of this (for affine costs) is in WFA (Marco-Sola *et al.* 2021) and its extension BiWFA (Marco-Sola *et al.* 2023) with reduced memory usage. Another key technique in accelerating global alignment is bitpacking, pioneered by Myers (1999). Rather than processing each DP cell individually, cost differences can be stored in word size w=64 bit vectors, allowing the processing of 64 DP states at once. This reduced the time complexity to O(n⌈m/w⌉), or O(n⌈s/w⌉) with banding. This bitpacking is implemented in the commonly used tool Edlib (Šošić and Šikić 2017). Modern CPUs can process more than 64 bits in SIMD registers (e.g. 256 bits for AVX2 or 128 bits for NEON). To effectively use parallelization, the DP matrix is often broken into smaller regions (Wozniak 1997, Farrar 2007, Liu and Steinegger 2023), allowing parallel processing such as in KSW2 (Li 2018, Suzuki and Kasahara 2018), BSAlign (Zhang *et al.* 2019), and SeqMatcher (Espinosa *et al.* 2025). Additionally, unlike for ASM, heuristics such as X-drop can be employed to reduce the search space (Altschul *et al.* 1990, Suzuki and Kasahara 2018, Liu and Steinegger 2023, Walia *et al.* 2024), while losing the guarantee that the best alignment is found. QuickEd (Doblas *et al.* 2025) first computes an approximate banded alignment and uses this as input for an exact alignment. A*PA and A*PA2 instead bound the search region by using A*, and retain the guarantee that an optimal alignment is found (Groot Koerkamp 2024, Groot Koerkamp and Ivanov 2024).

#### 1.2.2 Semi-global alignment

In *semi-global* alignment, a pattern *P* is aligned to a *substring* of a longer text *T*, and gaps at the start and end of *T* do not incur a penalty. Like in global alignment, only the alignment(s) with the lowest number of errors are reported. Semi-global alignment can either be between a short pattern and a much longer text (m≪n, e.g. searching a read in a reference genome), or between two sequences of similar length (m≈n, e.g. refining a mapped read). This approach was first introduced by Sellers (1979, 1980), and later termed *semi-global* by Gotoh (1999), who also termed *global* alignment. It is implemented in tools such as Parasail (Daily 2016), SeqAn (Reinert *et al.* 2017), Edlib (Šošić and Šikić 2017), and more recently Ish (Stadick 2025). Confusingly, the term semi-global is sometimes also used for different variants of alignment. Parasail (Daily 2016) uses it for all types of alignment that are not exactly global alignment, while Suzuki and Kasahara (2018) use it for *extension* alignment where the pattern has to match at the start of the text. When m≈n, semi-global alignment can benefit from adaptive banding methods as developed for global alignment, but this is not the case when m≪n. There, some methods (parasail, Seqan, Ish) simply compute the entire O(mn) DP matrix, while others (Edlib, Sassy) often only compute the top O(k) rows (Myers 1999). Thus, these two regimes lead to completely different algorithms.

#### 1.2.3 Cost models

A *cost model* defines what constitutes an *error* and the cost associated with each error. This concept originated in the early 1900s with systems designed to detect misspelled names by sound (Odell and Russell 1918). In the 1950s, Hamming distance was introduced for binary codes, measuring the number of differing bit positions (Hamming 1950), or in the context of DNA, the number of mismatches between two equal-length strings. Around a decade later, the Levenshtein distance was formalized (Damerau 1964, Levenshtein *et al.* 1966), which allows insertions and deletions alongside substitutions. Importantly, Levenshtein distance uses a *unit cost* model, assigning a cost of 1 to each edit, making it computationally efficient. However, this assumption is unrealistic for large insertions or deletions, as deleting or inserting long segments often represents a single biological event. This led to the development of gap-affine models, where gap opening and extension have different costs (Gotoh 1982, Altschul and Erickson 1986, Marco-Sola *et al.* 2021). For Sassy, we use unit-cost edit distance for its computational efficiency and the assumption that long indels are rare when aligning relatively short patterns.

#### 1.2.4 Approximate string matching

As mentioned before, the goal of ASM is to find all matches of a pattern *P* in a text *T* with ≤k errors (Galil and Giancarlo 1988, Navarro 2001). The key distinction from semi-global alignment is that not just the single best match should be reported, but that *all* matches with ≤k errors should be reported. We first discuss *streaming* algorithms, where the text is not known in advance, as opposed to algorithms that preprocess the text *T*. Moreover, we focus on the *k-difference* variant that uses edit distance rather than the *k-mismatch* variant that uses Hamming distance (Chhabra *et al.* 2025, Fiori *et al.* 2022, Gottlieb and Reinert 2025).

Searching exact matches of patterns became popular through algorithms such as Knuth–Morris–Pratt (Knuth *et al.* 1977) and Boyer–Moore (Boyer and Moore 1977). With the development of different cost models in the 1980s, algorithms were created to detect inexact matches with *k* errors. Initially, *ASM* described comparison of two strings (Ukkonen 1983, Hall and Dowling 1980), but later also described searching for a pattern as a substring of a text with ≤k errors (Landau and Vishkin 1985). Sellers proposed an O(mn) time algorithm (Sellers 1980), which was improved to $O(m^{2}+k^{2}n)$ (Landau and Vishkin 1985), and then O(kn) (Ukkonen 1985a). The introduction of bit-parallelism (Baeza-Yates and Gonnet 1992) led to complexities involving the word size *w*, such as O(k⌈m/w⌉n) (Wu and Manber 1992) in the famous agrep tool, O(mn log(σ)/w) (Wright 1994), and eventually O(⌈m/w⌉n) in Myers’ algorithm (Myers 1999). Later optimizations targeted specific scenarios: short patterns or small *k* (Baeza-Yates and Navarro 1999, Navarro and Baeza-Yates 1999, Bille 2012), fixed pattern lengths (Iliopoulos *et al.* 2001, Ho *et al.* 2018), and periodic texts (Cole and Hariharan 2002). Some were optimized for multi-pattern search (Muth and Manber 1996, Baeza-Yates and Navarro 1997), though here we focus on a single pattern.

Additionally, many algorithms leverage text preprocessing and indexing, such as text compression (Mäkinen *et al.* 2003, Kärkkäinen *et al.* 2000), suffix arrays (Landau and Vishkin 1986, Manber and Myers 1993, Huynh *et al.* 2004), and suffix trees (Ukkonen 1993). Others use pre-filtering with *n*-grams (Owolabi and McGregor 1988, Jokinen and Ukkonen 1991, Ukkonen 1992, Sutinen and Tarhio 1995, Bingmann *et al.* 2019), inexact hashing (Yao *et al.* 2010, McCauley 2021), heuristics (Koehn and Senellart 2010, Salmela *et al.* 2010), or search schemes on top of a bidirectional FM-index or move-index (Renders *et al.* 2022, 2024, Depuydt *et al.* 2024, Gottlieb and Reinert 2025, Renders *et al.* 2025). Such methods thus implement completely different algorithms than the streaming-based method that we focus on in this article, and are suitable for different applications.

#### 1.2.5 SIMD parallelism

Overall, the complexity of index-free methods did not improve beyond Myers’ O(⌈m/w⌉n). Practical speedups emerged with larger SIMD word sizes with W=256 or W=512 bits. Improvements then involved optimal utilization of *W*. For example, BGSA (Zhang *et al.* 2019) uses inter-sequence parallelism to compare multiple sequences to the same pattern, since intra-sequence parallelism is limited when m≤W (Zhang *et al.* 2019). An alternative approach is taken by A*PA2 (Groot Koerkamp 2024), where the dependency between SIMD lanes is broken by tiling them diagonally. Yet another approach is taken by SeqMatcher (Espinosa *et al.* 2025), where AVX-512 instructions are used to effectively use 512-bit integers. In contrast, Sassy splits the text into four chunks that are processed in parallel, somewhat similar to Farrar’s striped method (Farrar 2007) and as also used by SimdMinimizers (Groot Koerkamp and Martayan 2025), for a complexity of O(m⌈n/W⌉). This way, intra-sequence parallelism is maximized.

#### 1.2.6 Mapping

In modern applications, the text is often an assembled genome of many gigabases, and the number of patterns (reads) to be searched is very large. This means that index-free methods are infeasible, and in practice, *mappers* drop the guarantee to find all matches in favor of speed. Thus, we consider mapping to be approximate ASM. (ASM is *approximate* in the sense that matches are allowed up to *k* errors. Mapping is *approximate* in the sense that it is an approximate algorithm that does not guarantee to find *all* such matches.)

In the 1980s, with increasing sequence availability and the release of GenBank (Bilofsky *et al.* 1986), previous exact methods were no longer fast enough. Early mapping methods performed *exact* substring matches between the pattern *P* and database sequences, beginning with Wilbur and Lipman (1983) and followed by others using similar approaches (Lipman and Pearson 1985, Ning *et al.* 2001, Wu and Watanabe 2005) such as BLAST (Altschul *et al.* 1990).

Some methods controlled sensitivity based on the pigeonhole principle (Weese *et al.* 2012), while others tried to identify similar regions between *P* and the database sequences through spaced seeds (Ma *et al.* 2002, Rumble *et al.* 2009) or locality-sensitive hashing (Buhler 2001). As sequences got longer, the number of seeds also increased, leading to algorithms that reduced the number of seeds being stored, such as minimizers (e.g. Minimap2) (Li 2018, Jain *et al.* 2020), strobemers (e.g. StrobeAlign) (Tolstoganov *et al.* 2026), or by hashing subsequences instead of substrings (e.g. SubseqHash2) (Li *et al.* 2025).

However, in benchmarks these mappers do not detect all mapping locations: they can achieve over 99% sensitivity but not full coverage (Banović Deri *et al.* 2024), and their performance heavily depends on parameter settings such as the seed length (Oliva *et al.* 2021).

#### 1.2.7 Applications of ASM

The earliest methods for ASM, developed in the 1980s, proved directly useful for biological problems. For example, Myers (1986) used ASM to find a 16-nucleotide binding site of the LexA protein in a 48 kb virus genome. Today, ASM supports diverse applications, including read demultiplexing (Cheng *et al.* 2024), genome polishing (Tonkin-Hill *et al.* 2020), and CRISPR off-target searching (Chaudhari *et al.* 2020).

We focus on the latter due to its clinical relevance. CRISPR and its associated Cas proteins form an adaptive immune system in bacteria and archaea, evolved to defend against foreign nucleic acids such as bacteriophage and plasmid DNA (Mojica *et al.* 2009). In this system, foreign DNA is precisely cut using a template called single guide RNA (sgRNA). When the target DNA is flanked by a protospacer adjacent motif (PAM)—e.g. 5′-NGG-3′ in *Streptococcus pyogenes*—the CRISPR-Cas complex binds and cleaves the DNA, thereby neutralizing the invader. By modifying the sgRNA sequence, the CRISPR-Cas system can be programmed to cut virtually any DNA sequence. This technology has been applied to treat genetic diseases (Ledford 2019), enhance crop traits (Jaganathan *et al.* 2018), and engineer microorganisms (Shapiro *et al.* 2018). For an in-depth review, see Koonin and Makarova (2019). Notably, on 15 May 2025, CRISPR was used for the first time as a personalized treatment for a baby with carbamoyl phosphate synthetase 1 (CPS1) deficiency, a rare and life-threatening condition (Musunuru *et al.* 2025).

When CRISPR is engineered to target a specific sequence, it is crucial that no other, unintended sequences are cut. This is called *off-target* cutting. Hence, computational tools to screen for such off-target sites have been developed. These include Cas-OFFinder (Bae *et al.* 2014), CRISPRitz (Cancellieri *et al.* 2020), SWOffinder (Yaish *et al.* 2024), and CHOPOFF (Labun *et al.* 2025), with the latter two representing the current state-of-the-art.

While CHOPOFF is much faster than SWOffinder, it requires a time-consuming step of building an index before searching. Given that human genetic variation affects off-target profiles (Scott and Zhang 2017), we argue that with the advancement of personalized CRISPR therapies, there is a need for fast, index-free tools that are user friendly and robust to ambiguous bases.

## 2 Methods

We now describe our tool, Sassy. We start with some brief notation. Throughout the article, we assume that we are given a *pattern P* of length m:=|P|, and a *text* $T=t_{0}\ldots t_{n-1}$ of length n:=|T|, which are both strings over an alphabet Σ of size σ:=|Σ|. We write $T[i\ldots j]:=t_{i}\ldots t_{j-1}$ for a right-exclusive substring of *T*, and we use d(P,T[i…j]) for the edit distance between *P* and T[i…j]. We write $\mathrm{rev}(T):=t_{n-1}\ldots t_{1}t_{0}$ for the reverse of *T*, and for DNA sequences, we define the *complement* comp(T) as the sequence where each base is replaced by its complement (A↔T and C↔G, extended to IUPAC as well). The *reverse complement* is then rc(T):=rev(comp(T)).

### 2.1 Approximate string matching

We define AMS following Navarro (2001), but restrict ourselves to edit distance only.

Definition 1 (Approximate String Matching, ASM). Let *P* be a pattern of length m:=|P|, and let *T* be a *text* of length n:=|T|. Further, let $k\in N_{\geq 0}$ be the maximum number of errors allowed. The problem of approximate string matching, search(P,T,k), is to find all end positions j∈{0,…,n} in the text such that there exists an i∈{0,…,j} such that the edit distance d(P,T[i…j]) between *P* and T[i…j] is at most *k*.

**What is a match?** As defined above, a *match* is a position *j* in the text where an alignment of cost ≤k ends. In practice, one might rather care about all *substrings* of *T* that have edit distance ≤k to the pattern, i.e. all tuples (i,j) such that d(P,T[i…j])≤k (Sellers 1980, Definition 1). Or, even more exhaustively, one could consider all *alignments* of *P* to substrings of *T*, where an alignment is a specific sequence of edits transforming *P* into T[i…j].

In Sassy, we choose the first option: we find all end positions, and then do a *single* traceback for each of them.

**When do we have a match?** In practice, one is usually not quite interested in *all* matches. In particular, if there is an exact match ending in position *j*, all positions from j−k to j+k will have a cost ≤k (see Fig. 2). Thus, there are numerous options for which matches to report:

1. **All**. Report (matches ending in) *all* end positions with cost ≤k.
2. **Single best**. Report only a *single best* end position (if ≤k). Supported by Seqan.
3. **All best**. Report *all* positions where a match of *globally optimal* cost ends (if ≤k) as done in semi-global alignment as defined by Sellers (1980) and supported by Edlib and Seqan.
4. **Non-overlapping**. Report only end positions that are at least (roughly) *m* apart.
  In Sassy, we take a different approach, that we argue is more principled:
5. **Local minima**. Report only *rightmost local minima* ≤k.

This is similar to the idea of Sellers (1980) to report all substrings T[i…j] that cannot be shrunk or grown into a substring with lower edit distance, with the difference that we only report end positions, and that we only report a single match for each plateau of local minima.

**ASM is not reverse-invariant.** We note here that when reporting end positions, it is typically hard to guarantee that the matches reported by search(P,T,k) are in one-to-one correspondence with those reported by search(rev(P),rev(T),k), since the number of (local/global minima) end positions ≤k can differ in the forward and reverse case, as exemplified in Fig. 2. Reporting *all* matching substrings T[i…j] would avoid this, but neither Sassy nor other tools do this in practice.

When searching reverse complements is enabled, Sassy  *is* invariant to reverse complements of the text: we search *P* in *T* and rc(T), so that both searches are in the natural direction of the pattern and searching *P* in rc(T) and rc(rc(T))=T gives the same result. (As an implementation detail, we actually search comp(P) against rev(T), so that we can avoid taking the complement of *T*. Invariance under complements is trivial.) Searching rc(P) in *T* or rc(T)  *would* change the set of matches.

**Traceback.** Given the set of end positions that define a match, we can run a traceback from each of them to obtain both the position in the text where the match starts, and the corresponding alignment. Sassy simply recomputes the part of the DP matrix preceding each end position and traces back through that. The traceback greedily chooses matches and substitutions if possible, and then falls back to deletions and insertions, in that order.

### 2.2 Bitpacking and SIMD tiling

**Figure 1:**
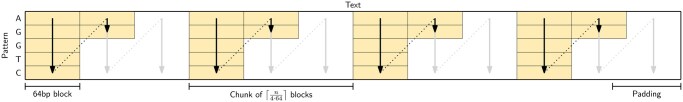
The tiling strategy used by Sassy. The text is first split into word-size blocks of 64 bases. Then, the list of blocks is split into four chunks that are processed in parallel, with one SIMD lane per chunk. The text is implicitly padded as needed. Within a chunk, filling the matrix proceeds block-by-block. For each block, all (up to, see Section 2.3 and Fig. 3) m=|P| rows are computed before proceeding to the next block. Each chunk is extended into the succeeding chunk as long as there is a sufficiently good “in progress” alignment (not shown).

Figure 1 shows how Sassy applies both Myers’ bitpacking (Myers 1999) and SIMD. Using bitpacking, a *block* of w=64 states of the DP matrix can be computed in parallel. Whereas other methods typically tile these bitvectors in the direction of the pattern, we tile them in the direction of the text. This way, SIMD lanes process different parts of the text, ensuring speedups even for short patterns. Consequently, the bitpacking is also in the text direction to allow for efficient *profile* lookups (Appendix C, available as supplementary data at *Bioinformatics* online). Pseudocode of the main search function of Sassy can be found in Appendix A, available as supplementary data at *Bioinformatics* online.

**Figure 2:**
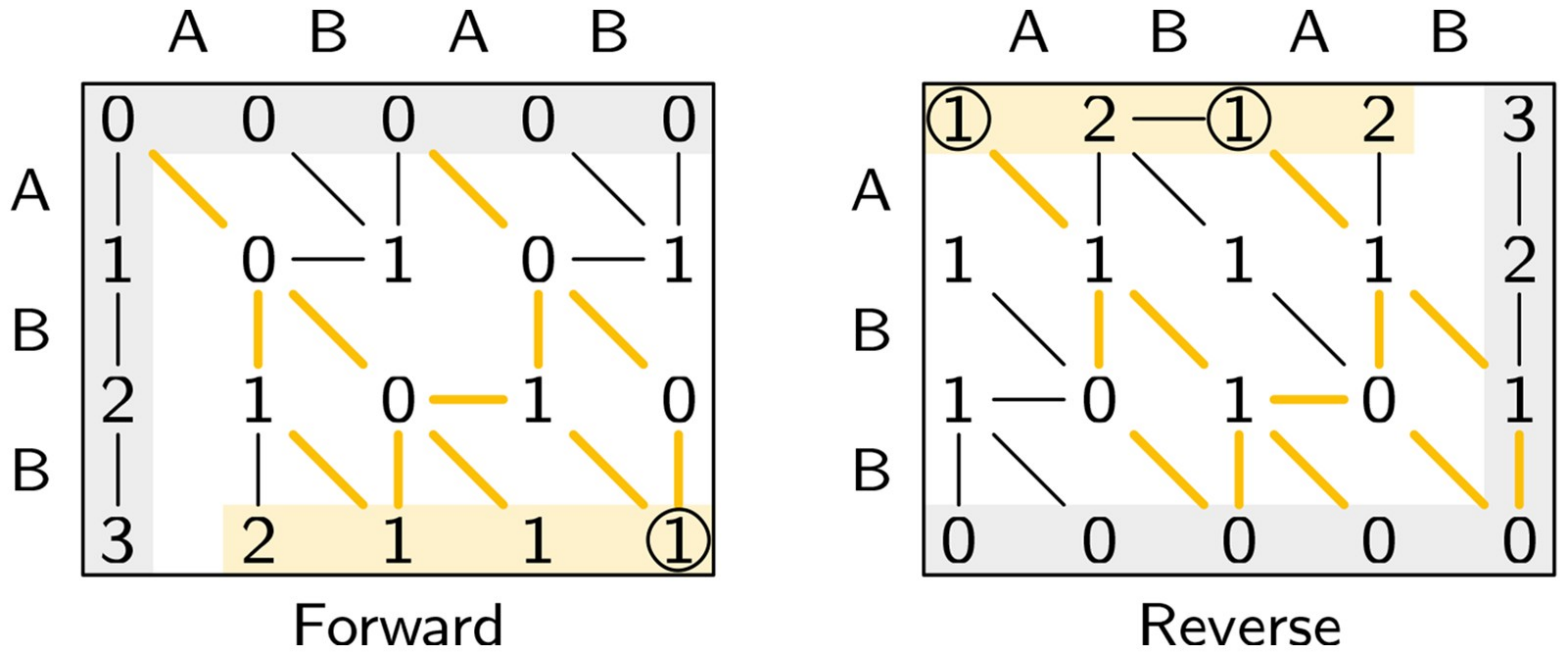
Approximate string matching. An example of finding all occurrences of ABB in ABAB. On the left, the forward search initializes the top and left of the matrix (shaded in grey). Then, it shows all optimal paths to each state. On the bottom, the final distances are highlighted, and all optimal alignments of cost 1 are highlighted in yellow. By default, Sassy only starts a trace in the circled 1, a rightmost local minimum. The right figure shows the reverse alignment, where the matrix is filled from the bottom right to the top left. Note that the set of optimal alignments is the same, but that the number of local minima (1 versus 2) and global minima (3 versus 2) both differ.

**SIMD.** We use 256 bit SIMD widths using either AVX2 or two parallel NEON registers to compute four *lanes* of 64 bit words in parallel. (Using AVX-512 did not provide further gains since iterating eight text chunks in parallel became a bottleneck.) We avoid dependencies between SIMD lanes by splitting the text into four chunks of ⌈n/256⌉ blocks each. Each lane then processes one chunk: we iterate over the 64-character blocks of each chunk, and for each block compute the *m* rows of the matrix.

As with Farrar’s striped method (Farrar 2007), there may be some “patching up” to do when a good alignment crosses the boundary between two chunks. In our case, we extend each chunk to the right (overlapping with the next chunk) as long as there is a partial alignment of cost ≤k that started inside the original chunk. Especially when the text is long (so that ⌈n/W⌉≈n/W), this intra-sequence parallelism is near-optimal.

### 2.3 Early break

**Figure 3:**
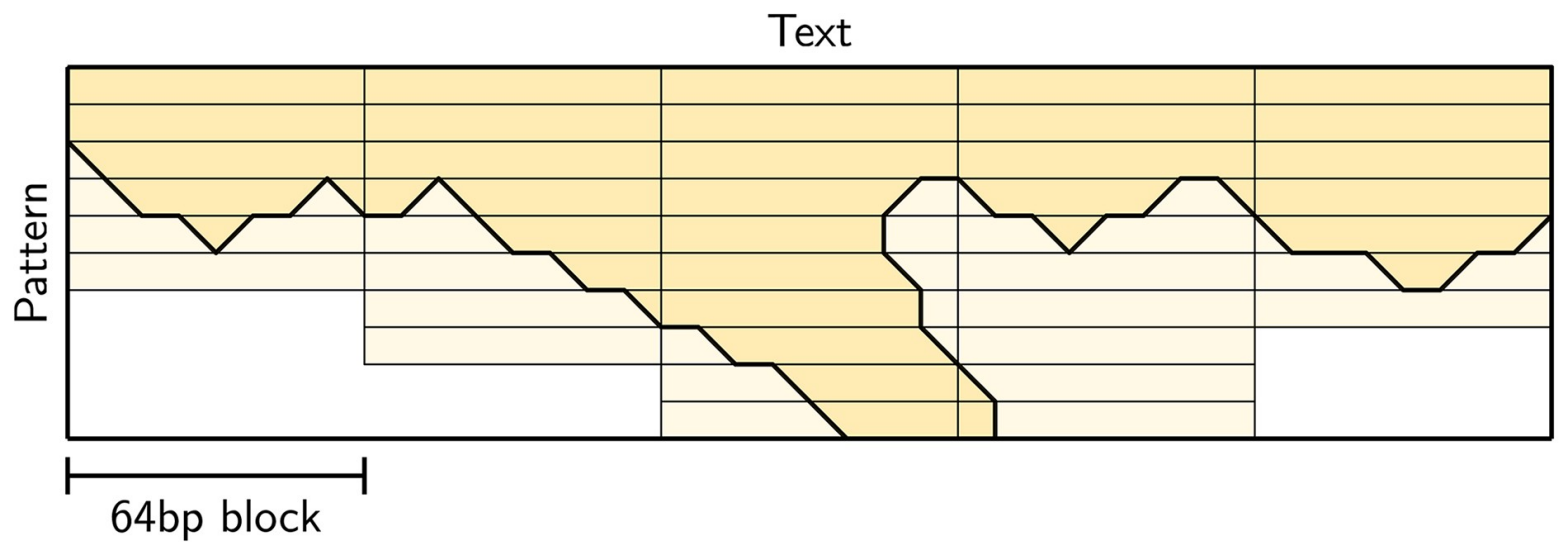
Early break. We are only interested in entries of the DP matrix with value ≤k, as shown in the bold-outlined area. As soon as all entries in a row are >k, we can stop processing that block of text, as in the first and second block. Then, we only reach the bottom of the matrix when matches are present, as in the third block. One exception is shown in the fourth block: when there are states at the end of the previous block at distance ≤k, we must continue at least one row beyond that point. Since we use SIMD to process four chunks in parallel (not shown here), in practice we continue until the values in all four lanes are >k.

The complexity of computing the entire DP matrix as described so far is O(m(⌈n/W⌉+⌈m/w⌉)), where the final +⌈m/w⌉ accounts for overlaps between chunks. In ASM, we only care about matches with a cost at most *k*, and thus, parts of the DP matrix where values are >k can be skipped (Ukkonen 1983, 1985b, Myers 1986), as shown in Fig. 3. In particular, the edit distance between two uniform random and equal-length DNA sequences is typically around 45% of their length, so that most of the time, the cost of aligning a prefix of length 2·k of the pattern already incurs a cost >k. More formally, Chang and Lampe (1992) proved that the number of states with cost ≤k is O(kn) when searching a random text.

Thus, as soon as all *W* columns corresponding to a SIMD vector contain a value >k, and additionally there are no remaining states with cost ≤k at the end of the preceding blocks, we can stop with the current four blocks and move on to the next.

### 2.4 Library and command line tool

The main entrypoint of the Sassy Rust library is a function search(pattern, text, k) that returns one match for each rightmost local minimum endpoint. It can optionally return matches for *all* endpoints, and supports reverse complements. The input can be either (case insensitive) ASCII text, simple ACGT DNA, or more general IUPAC-encoded DNA where sequences (*both* the pattern and the text) may contain bases such as N (matching ACTG), Y (matching CT), and R (matching AG). This is handled by selecting a *profile* (Appendix C.1, available as supplementary data at *Bioinformatics* online). In case of simple DNA, we provide a function to validate that no non-ACGT bases are present. We provide both C and Python bindings.

We also provide a simple command line tool for searching a sequence in all records of a Fasta file, that can be installed via cargo install sassy or conda install -c bioconda sassy. Examples of commands are:

sassy search ––alphabet dna ––no-rc -k 0 ––pattern CAT data.fa

sassy search ––alphabet iupac -k 1 -f patterns.fa genome.fa.gz

sassy crispr -k 5 ––guide guides.fa ref.fa

The first searches for an exact match of CAT in all records of data.fa. The second searches each record of patterns.fa in genome.fa.gz, while allowing up to 1 error and also searching the reverse-complement text. The last searches each of the guides in guides.fa against ref.fa while allowing at most five errors. Here, PAM sequences must match exactly and the preceding sgRNA can contain up to five errors.

Whereas the library is single-threaded, the command line tool maintains a queue of (P,T) tuples that are distributed (in batches) over all threads. Further details on the implementation can be found in Appendix C, available as supplementary data at *Bioinformatics* online.

## 3 Results

We compare Sassy against Edlib (Šošić and Šikić 2017) (which uses bitpacking but not SIMD) in two metrics: the throughput of searching random DNA sequences without matches (the number of text bases processed per second), and the throughput of finding and tracing matches (the number of matches that can be found and processed per second). Section 4 shows specific applications of Sassy. The code and data for the benchmarks can be found in the evals directory at https://github.com/RagnarGrootKoerkamp/sassy. These experiments were run on an Intel Core i7-10750H with 6 cores, 12 threads, AVX2 support, and running at a fixed frequency of 3.6 GHz.

### 3.1 Throughput of text searching

**Figure 4:**
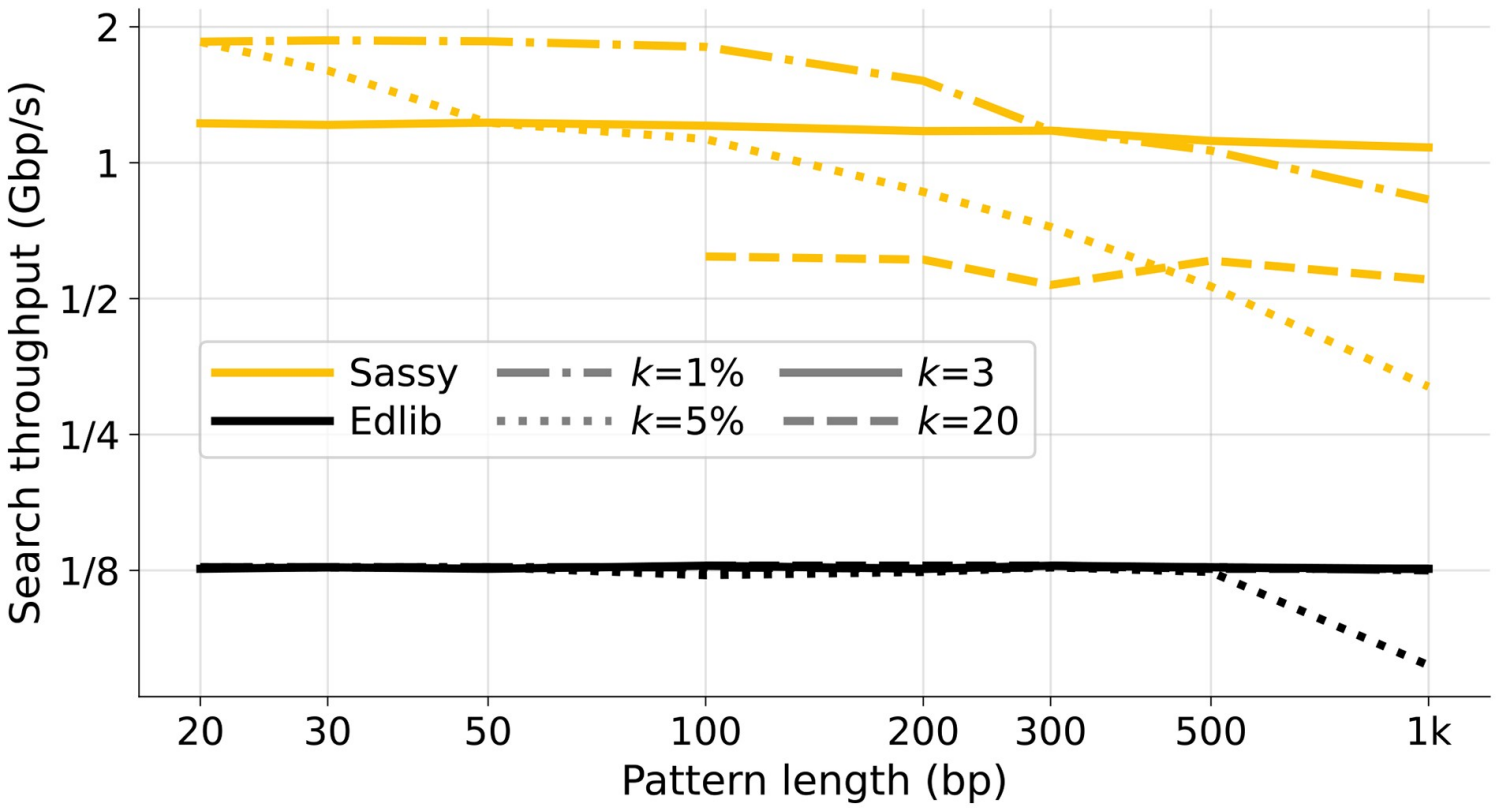
Throughput of searching patterns of varying length. The pattern length *m* (*x*-axis) ranges from 20 to 1000, and the error threshold *k* (line style) is either fixed at 3 or 20, or computed as ⌈m/100⌉ or ⌈m/20⌉. Only points with m>3k are shown to avoid spurious matches. All points are computed by averaging over 1000 random patterns and texts of length $n=10^{5}$, and then converting to throughput. Note that this does not include searching the reverse-complement strand. Sassy has up to 10× higher throughput than Edlib when *k* is small.

**Figure 5:**
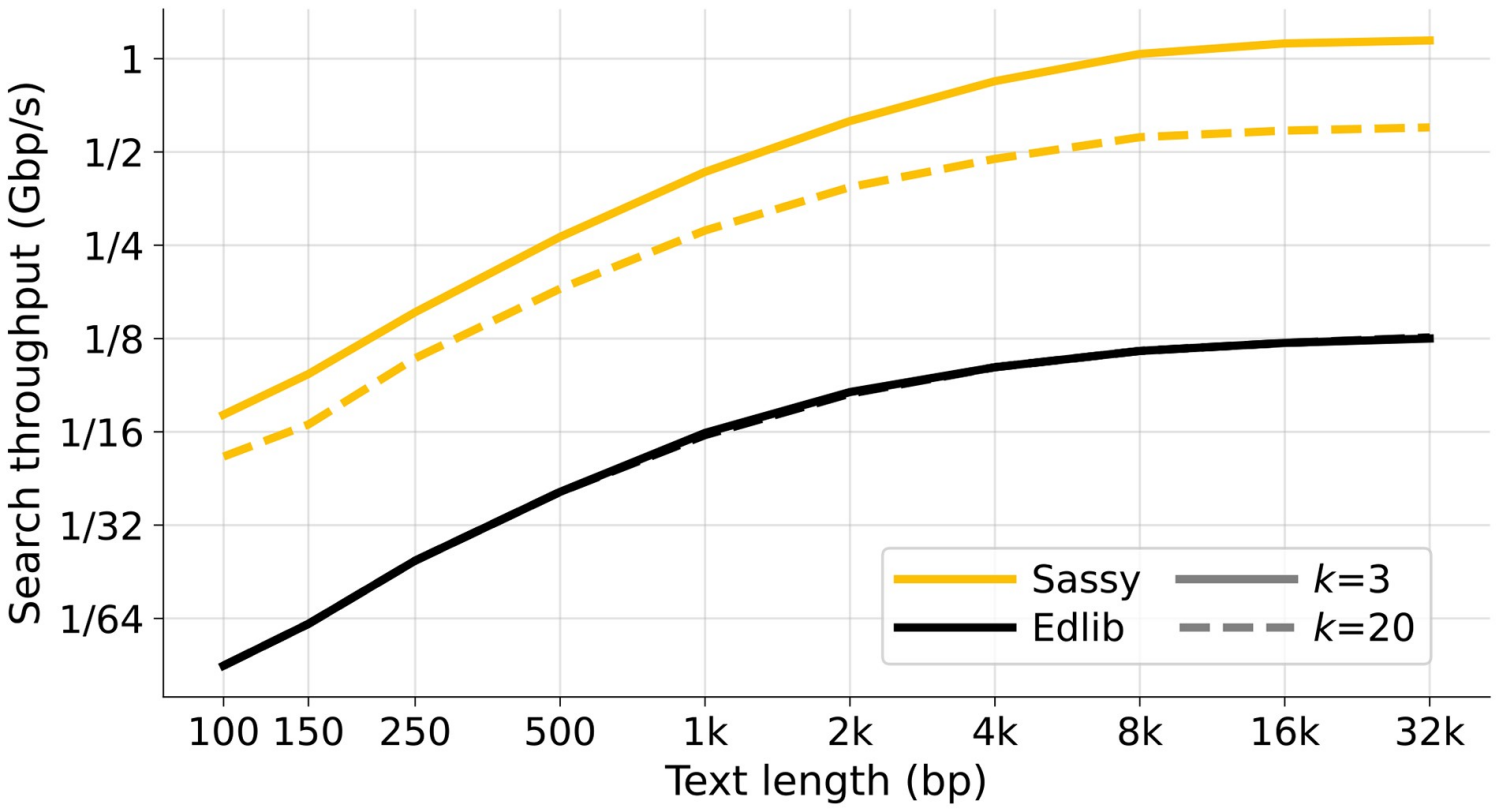
Throughput of searching texts of varying length. We search a pattern of length m=100 against texts with length varying from n=100 to n=32000 bp, with k∈{3,20}. All points are computed by averaging over 1000 random texts and then converting to throughput. Note that this does not include searching the reverse-complement strand. While Sassy is consistently faster than Edlib, its relative advantage is smaller for shorter texts.

In Figs 4 and 5, we compare the text throughput of searching with Sassy and Edlib. Each data point is the average of searching 1000 random DNA patterns of length *m* in a random text of length *n*. Figure 4 compares searching patterns of varying length (20≤m≤1000) in a long text ($n=10^{5}$) for varying error thresholds (0≤k≤50=0.05·1000), while in Fig. 5, a pattern of length m=100 is searched in texts of length 100≤n≤32000 for k∈{3,20}.

We exclude Seqan, since it is consistently outperformed by Edlib. A comparison against parasail, an affine-cost aligner, can be found in Appendix E, available as supplementary data at *Bioinformatics* online. It is around 10× slower than Edlib and 100× slower than Sassy. Ish is an up to 35% faster re-implementation of parasail, but does not provide Rust bindings. Lastly, other tools such as agrep do not accept FASTA input.

Sassy is faster across all *m*, *n*, and *k*. For short patterns (m≤50 bp), Sassy has throughput over 1.2 Gbp/s whereas the throughput of Edlib does not exceed 130 Mbp/s. Since both the pattern and text are random, no matches occur, and the early break causes Sassy to have complexity O(k⌈n/W⌉). Indeed, for constant *k* the throughput is independent of *m*, while it decreases when k=0.05·m. Edlib, on the other hand, has throughput nearly independent of *k*: in nearly all cases we have k≤20, so that crossing the first w=64 rows of the DP matrix already incurs a cost >k. This matches the complexity of O(⌈k/w⌉n). In Fig. 5, we see that both Edlib and Sassy are faster when searching longer texts and have large constant overhead when searching small texts, in case of Sassy due to relatively large overheads between text chunks. At text length n=150, Sassy is 4× to 6× faster than Edlib, which increases to 5×  9× speedup for longer texts.

The throughput when searching ASCII (slightly faster) or IUPAC (slightly slower) text is within 5% compared to DNA.

### 3.2 Affine-cost aligners

Sassy is over 10× faster than tools implementing affine-cost alignments. For example, Sassy needs 4.5 s to search a pattern of length 23 in a human genome (with up to k=4 errors, excluding searching the reverse-complement). For the same task, parasail (Daily 2016) in semi-global mode with default cost parameters takes 53–69 s, depending on the exact configuration (8 or 16 bit values, and SSE4.1 versus AVX2), reporting up to 1.45 GCUPS (giga cell updates per second). Ish (Stadick 2025) with default parameters takes 69 s (SSE4.1) to 110 s (AVX2). Running parasail with the same costs as Ish takes 81–198 s. These methods are slower both because they store larger values (instead of bitpacking), and because they compute two additional *affine layers* of the DP matrix.

We propose that exact affine-cost alignment could be achieved by using a fast edit distance alignment to identify candidate regions.

### 3.3 Throughput of tracing

**Figure 6:**
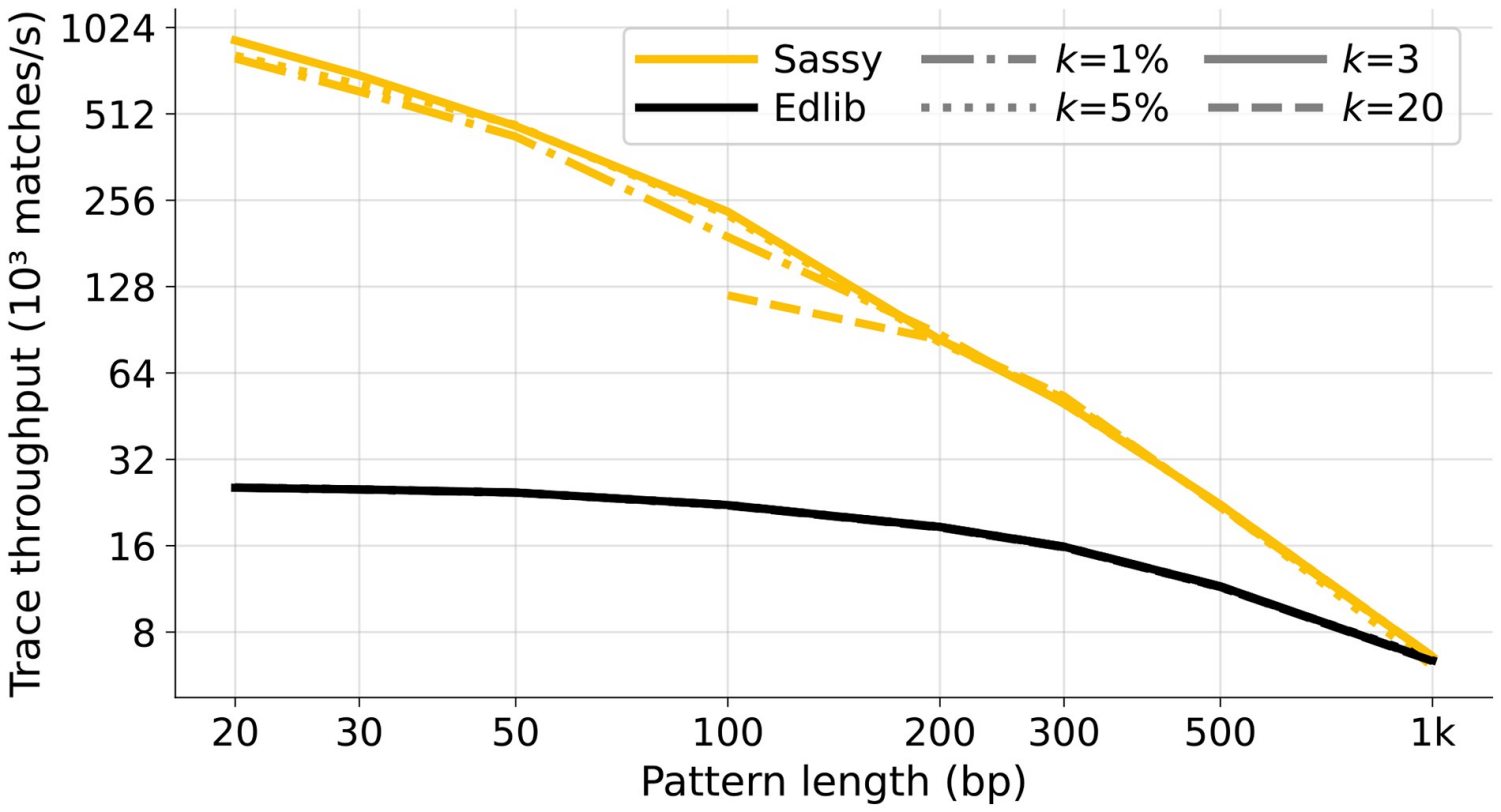
Throughput of finding matches. When the text contains a match, this causes a larger part of the matrix to be computed since the early break does not trigger. Later, this part of the matrix is recomputed in full and stored in $O(m^{2}/w)$ words of memory so that a traceback can be done. To measure the total time it takes to process a match, we use the same setup as in Fig. 4 with the addition of a single copy of the pattern planted at the end of the text. We measure the time difference with the version without match and report the corresponding throughput as the number of matches that can be processed every second.

In Fig. 6, we show the throughput of finding matches. This includes the time to locally compute all rows of the matrix (rather than just the top O(k) rows), the time to recompute the matrix region containing the match, and the time for tracing through the filled matrix. We use the same setup as in previous experiments, and “plant” a single match at the end of the text. We then compare the run time of the same text with and without match.

Placing the match at the end avoids triggering the dynamic reduction of *k* in Edlib: Since Edlib performs semi-global alignment and only searches match with the minimum edit distance, it reduces *k* whenever it finds a match with cost <k. If the match was placed earlier in the text, this reduction would confound the measurement by timing both the tracing time and its *k* reduction strategy. By placing the match at the end of the text, we largely isolate the tracing cost from the *k* reduction strategy.

For short patterns, Sassy is over 10× faster per match than Edlib. For longer patterns, Sassy’s throughput goes down quadratically as it naively computes the $O(m^{2}/w)$ words to fill the part of the matrix where a match is. Edlib does not slow down as much, likely due to O(ns) banding, but is nevertheless still slower than Sassy for patterns up to length m≤1000.

## 4 Application: finding CRISPR off-targets

Finding short sequences has many important applications, with CRISPR off-target searching being of particular recent interest (Musunuru *et al.* 2025). SWOffinder (Yaish *et al.* 2024) and CHOPOFF (Labun *et al.* 2025) are currently among the fastest and most accurate tools for identifying off-target sequences. We extended Sassy with CRISPR off-target searching, enabling the search for PAM motifs—such as the Cas9 NGG motif—preceded by a guide RNA (gRNA) sequence (Ran *et al.* 2013).

Unlike SWOffinder, sassy crispr reports at most one match for every location where the PAM has an exact match.

### 4.1 Searching 61 guide RNAs in the human genome

First, we briefly summarize the algorithms used by the benchmarked tools. SWOffinder uses Smith–Waterman alignment to fill the entire m×n matrix, identifying all end positions with ≤k edits. It then filters these alignments to only those with at most one indel (Yaish *et al.* 2024). CHOPOFF takes a different approach by first indexing all PAM locations in the target genome and storing their prefixes, i.e., the sgRNA flank. Then they precompute all the edit distance paths, within *k*, through these prefixes. This allows instant lookup of sgRNA sequences with at most *k* edits of previously identified PAM sites. These indexes range from 3.2 Gbp (k=0) to 4.5 Gbp (k=4) for the human genome. The prefix edit paths are shipped with CHOPOFF for k≤4. However, computing this for k=5 did not finish in 10 h. Sassy’s algorithm is similar to its regular search, but with an additional filter prior to traceback: when the user requests the PAM sequence to be unmutated, then the traceback is only performed when the exact PAM is present.

To compare Sassy to the other off-target search tools, we used the benchmark from the CHOPOFF paper (Labun *et al.* 2025). This searches 61 guide sequences with the NGG PAM against the human genome. Experiments were run on an Intel(R) Xeon(R) Gold 6240, using 16 threads for each job. Results are in Table 1.

**Table 1:** Time (mm:ss) to search 61 sgRNAs in Human genome CHM13 using 16 threads, and number of matches.^a^

	Sassy		CHOPOFF		SWOffinder	
*k*	Time	Matches	Time	Matches	Time	Matches
0	0:19	62	0:18 (19:47)	62	40:21	62
1	0:20	127	0:19 (23:53)	127	42:07	155
2	0:23	1284	0:19 (23:59)	1284	39:54	1411
3	0:28	32 033	0:26 (23:39)	32 033	40:34	35 434
4	0:30	405 401	2:23 (23:56)	405 401	41:38	471 395
5	0:44	4 093 387	–	–	40:43	1 476 640

a For CHOPOFF the time to build the index is shown in parentheses, and was terminated after 10 h for k=5. All tools require exact matches of the 3 bp PAM sequence. SWOffinder reports more matches for small *k* because it searches with the PAM sequence at the *front* of the pattern, sometimes resulting in multiple matches for each match of the PAM. For k=5, it has *fewer* matches, because it only allows each match to have up to one indel.

For large values of *k*, Sassy outperforms both competitors by a wide margin. In fact, for k≥4, Sassy is more than 100× faster than SWOffinder and over 4× faster than the index-based CHOPOFF. Notably, Sassy completes the k=5 search in just 44 s, whereas CHOPOFF’s index building for k=5 exceeded 10 h (and was therefore omitted). For smaller values (k≤3), Sassy and CHOPOFF have comparable performance, with Sassy trailing by only a few seconds. We do note that CHOPOFF is faster when there are substantially more sgRNA patterns, as most time is spent on loading the index, which is not parallelized over multiple threads.

We note that full support for IUPAC bases, as Sassy and CHOPOFF have, is important for this application, since human genome assemblies may not always be fully resolved—see Appendix D, available as supplementary data at *Bioinformatics* online.

Thus, Sassy is an extremely fast tool that does not require building an index, making it ideal for personalized, reference-free, off-target screening.

## 5 Discussion

Sassy solves ASM, and allows fast searching for short DNA sequences without using an index. The main algorithmic novelty is to use *horizontal* bitpacking of deltas, and intra-sequence parallelism using SIMD (Fig. 1), leading to a complexity of O(k·⌈n/W⌉) when searching random text. This improved complexity allows searching text at nearly 2 Gbp/s, and up to 15× speedup over Edlib.

Practically speaking, Sassy is a simple-to-use tool with many applications. Since Sassy is index-free, it easily supports IUPAC characters in both the pattern and text. It is significantly faster than other index-free methods for searching CRISPR off-target matches, and is being integrated into other tools such as CRISPRapido (https://github.com/pinellolab/crisprapido), which uses Sassy as a pre-filter for off-target detection with a more fine-grained (affine-cost) cost model, and Barbell (Beeloo *et al.* 2025), a demultiplexer for Nanopore reads.

It is left to the user to choose a suitable edit distance threshold *k* that captures all biologically relevant matches, and to post-process and/or refine the matches with a more accurate affine or position-specific cost model if needed.

### 5.1 Limitations and future work

When the text that is searched is short (n≤1000 or so, and in particular for n=150), Sassy fails to reach its maximum throughput (Fig. 5), because the overhead of initializing each search is relatively large and there is a lot of overlap between adjacent text chunks. This is particularly relevant when searching barcodes in reads, and motivates ongoing work on Sassy2 (Beeloo and Groot Koerkamp 2026), which searches batches of multiple patterns at once.

Sassy is primarily designed to search patterns with length m≤100 or so, and includes a quadratic $O(m^{2}/w)$ component in both the initial filling of the matrix (Fig. 3) and the traceback, whereas a banded O(mk/w) approach would be sufficient in theory.

Lastly, Sassy only provides limited benefit when a *static* text is searched with many (at least 100–1000) patterns: in that case, search schemes for ASM (Renders *et al.* 2022, 2024, Gottlieb and Reinert 2025) that use a bidirectional index, such as Columba (Depuydt *et al.* 2024, Renders *et al.* 2025), should be preferred instead.

## Supplementary Material

btag244_Supplementary_Data

## Data Availability

The code and data for this article are available on Github at https://github.com/ragnarGrootKoerkamp/sassy.
