# Supplementary material for: Sassy: fuzzy searching DNA sequences using SIMD
Source: Bioinformatics. 2026 May 24;42(5):btag244. doi: 10.1093/bioinformatics/btag244 (PMC13202463; doi:10.1093/bioinformatics/btag244)
Supplement: btag244_Supplementary_Data [file btag244_supplementary_data.pdf]

A. Pseudocode

Alg. 1: Pseudocode for Sassy's main Search function, that takes as input the pattern P of length m and text T of length n, and returns a list of end-positions in the text where alignments of cost $\leq k$ end. Some of the operations operate on SIMD registers such as $[0,0,0,0]$. ComputeBlock applies Myers' bitpacking algorithm to SIMD registers. Details of Eq, AllAboveK and FindEndPositionsAtMostK can be found in Appendix C.

1: **function** Search(P, T, k)
2: $B \leftarrow \lceil n/(4\cdot 64)\rceil$ ▷ *Number of 64bp blocks per chunk.*
3: $j_{\leq k} \leftarrow 0$ ▷ *Last row in previous block ending in a value $\leq k$.*
4: $j_{\max} \leftarrow 0$ ▷ *Last computed row in previous block.*
5: $v^+ \leftarrow [[1,1,1,1];m]$ ▷ *Initialize list of SIMD of +1 deltas.*
6: $v^- \leftarrow [[0,0,0,0];m]$ ▷ *Modified when $\alpha < 1$ for overhang.*
7: $M \leftarrow [[],[],[],[]]$ ▷ *Matches (pos, cost) per lane.*
8: **for** $i \in \{0,\dots,B+\lceil(m+k)/64\rceil-1\}$ **do**
9: $i_{\max} \leftarrow i$
10: $h^+, h^- \leftarrow [0,0,0,0]$ ▷ *Deltas in current row.*
11: $d_s, d_e \leftarrow [0,0,0,0]$ ▷ *Dist to start and end of each block.*
12: $j'_{\leq k}, j'_{\max} \leftarrow 0$
13: **for** $j \in \{0,\dots,m-1\}$ **do**
14: $d_s \leftarrow d_s + v^+[j] - v^-[j]$
15: **for** $\ell \in \{0,1,2,3\}$ **do**
16: ▷ *Profile determines a bitmask indicating equal chars.* ◁
17: $eq[\ell] \leftarrow \mathsf{Eq}(P[j], T[B\cdot\ell+64\cdot i\dots B\cdot\ell+64\cdot i+64])$
18: ComputeBlock$(v^+[j], v^-[j], h^+, h^-, eq)$ ▷ *Myers bitpacking.*
19: $d_e \leftarrow d_e + v^+[j] - v^-[j]$
20: **if** $\min_\ell d_e[\ell] \leq k$ **then**
21: $j'_{\leq k} \leftarrow j$
22: $j'_{\max} \leftarrow j$
23: ▷ *Check if all 256 values are $> k$.* ◁
24: **if** $j > j_{\leq k}$ and AllAboveK(d_s, h^+, h^-, k) **then**
25: **for** $j' \in \{j+1,\dots,j_{\max}\}$ **do**
26: $v^+[j'] \leftarrow [1,1,1,1]$ ▷ *Reset "dirty" skipped rows.*
27: $v^-[j'] \leftarrow [0,0,0,0]$
28: $j_{\max} \leftarrow j'_{\max}$
29: $j_{\leq k} \leftarrow j'_{\leq k}$
30: **if** $i \geq B$ and $64\cdot(i-B) - j > k$ **then**
31: Goto 35, break out of i. ▷ *Done with chunk overlap.*
32: Goto 8, continue with next i ▷ *Early break.*
33: ▷ *Find (local minima) end positions with cost $\leq k$.* ◁
34: FindEndPositionsAtMostK(M, d_s, h^+, h^-, k)
35: **for** $\ell \in \{1,2,3\}$ **do** ▷ *Prune duplicate matches in overlap.*
36: $M[\ell] \leftarrow \{x \in M[\ell] : x \geq B\cdot\ell + 64\cdot(i_{\max}+1)\}$
37: **return** M

B. Overhang alignments

In some applications, it is useful to not only find occurrences of the pattern that are fully contained in the text, but also those that extend beyond the text. For example, this is the case for reads that may only partially contain a barcode, or in the case of fragmented assemblies [Abramova et al., 2024]. Following Abrahamson [1987], we call these *overhanging* matches.

Definition 2 (Overhanging match) Given pattern P of length m and text T of length n:

1. there is a *left-overhanging* match when a suffix $P[l\dots m]$ matches a prefix of T,
2. there is a *right-overhanging* match when a prefix $P[0\dots m-l]$ matches a suffix of T.

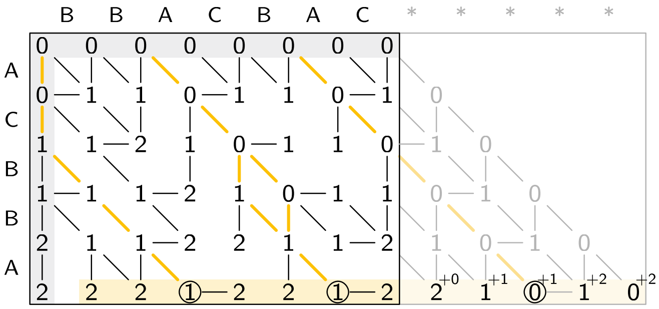

Fig. 7: Overhang alignment of ACBBA against BBACBAC, with overhang cost $\alpha = 0.5$. On the left, the state in row j is initialized with cost $\lfloor j\alpha\rfloor$, and a left-overhanging alignment (highlighted) is found where BBA matches and AC extends beyond the text for a cost of $\lfloor 2/2\rfloor = 1$. On the right, the text is padded with m wildcard symbols, so that the costs on the right side of the matrix are replicated in the bottom row. Then, for each extended state in columns $i > n$, $\lfloor(i-n)\alpha\rfloor$ is added to the cost. This finds a right-overhanging alignment where AC matches and BBA extends beyond the text for a cost of $\lfloor 3/2\rfloor = 1$.

In either case, each of the l overhanging (unmatched) characters of P incurs a cost of $0 \leq \alpha \leq 1$, for a total *overhang cost* of $\lfloor l\cdot\alpha\rfloor$.

Figure 7 shows an example with $\alpha = 0.5$ (Sassy's default) with three matches of cost 1: a left-overhanging match, a non-overhanging match, and a right-overhanging match. When $\alpha = 1$, this corresponds to semi-global alignment and ASM, whereas $\alpha = 0$ corresponds to *overlap* alignments.

Sassy only needs a slight modification to find overlapping matches: the left of the DP matrix is initialized with cost $\lfloor i\alpha\rfloor$ instead of i, and on the right we extend the text with m "wildcard" symbols, so that the costs in the right column of the matrix are "copied" to the bottom row. Then, we manually add the overhang cost for those extended states. In practice, we use the IUPAC alphabet for this, and simply append N characters.

C. Implementation Notes

We now provide some more details on the functions used in Algorithm 1.

AllAboveK(d_s, h^+, h^-, k)**.** This function takes as input the distance d_s to the start of 4 blocks, a threshold k, and the bit-encoded horizontal differences in each block. It then checks if the represented values in all blocks are $> k$. In practice, this function is slightly slow, and we call it not every row (as shown in the pseudocode), but only at most every 8 rows.

It processes each lane ℓ independently. First, we *pack* the bits of $h^+[\ell]$ and $h^-[\ell]$ into p (using `_pext_u64` available in BMI2) to remove positions where the delta is 0 and both have an indicator bit of 0. Then, a 1 in p indicates -1 and a 0 indicates $+1$ (with $+1$ padding at the end). We split the 64-bit value p into bytes, and do a precomputed table lookup that gives the total delta across the byte, and the minimum prefix sum in the byte. Then we do a rolling sum over the bytes to compute the minimum overall prefix.

FindEndPositionsAtMostK(M, d_s, h^+, h^-, k)**.** This function again works lane-by-lane. Unlike AllAboveK, this function is only called at most once per block of text, when the iteration over j reaches the end of the pattern. Thus, it is less performance critical, and we implement it by simply iterating through the 64 bits of the

bitmasks and keeping a rolling sum for the current score in each column. Then, each time a value $\leq k$ is seen, the index of the text and the corresponding cost are pushed to the list of matches.

C.1. Profiles

The job of a *profile* is to take a single character $P[j]$ of the pattern and a slice of 64 text characters, and determine a bitmask $\mathsf{Eq}(P[j], T[x \dots y])$ indicating which of the text characters equal $P[j]$ [Rognes and Seeberg, 2000].

We recommend having a look at the code, in the `src/profiles` directory of the git repository. Currently we only support AVX2, and thus, this is subject to change.

ASCII. For the ASCII profile, we implement this as follows. For each block of text, we precompute a 256-long array of 64-bit words, so that the mask for each ASCII character of the pattern can simply be looked up. The array is filled by using 256-bit SIMD instructions to compare each byte up to 256 to both the first 32 characters (`[u8; 32]` is 256 bits) and last 32 characters of the text slice, and merging the two 32-bit values.

For efficiency, we first compute a list of all distinct bytes in the pattern, and then only fill table rows corresponding to those bytes.

Case-insensitive ASCII. In this case, we first lowercase all text and pattern characters before doing the equality check. This is done by xor'ing the value of all uppercase bytes by 32.

DNA. DNA only has 4 characters, and so we precompute a table of size 4. Each ACTG character is encoded into an integer in $\{0, 1, 2, 3\}$ by first shifting right 1 bit and then only looking at the bottom 2 bits.

Optionally, it can first be checked that the text only contains valid bases in ACTG. This is done by ensuring that each position case-insensitively equals one of ACTG.

IUPAC. Here, we start by building a table that maps each IUPAC character to a 4-bit mask indicating which subset of ACTG it matches. Since only letters are allowed as input, it is sufficient to only consider the low 5 bits of each input character leaving 32 possible values. This automatically collapses upper and lower case values. We would now like to use a `[u8; 32]` SIMD register as a lookup table (via shuffle instructions), but unfortunately cross-128-bit lane byte shuffles are not supported on AVX2. We work around this: each byte only contains 4 bits of data, and thus, we can merge them, so that byte i in a `[u8; 16]` contains the 4 bits of both i (low half) and $i+16$ (high half). Then, we can use this as a lookup table on the low 4 bits of each text character, and use the 5th bit to select either the low or high half of the returned byte.

From here, we proceed similarly to before: we first build a list of characters occurring in the profile. Then we encode each character to its 4-bit representation, and find the text characters that this "intersects" with.

D. Support for ambiguous bases

Depending on their quality, human genome assemblies can contain over 10% ambiguous bases, as seen in GRCh38 [Nurk et al., 2022]. In search applications with clinical implications, such as CRISPR off-target analysis, it is crucial to report matches in regions containing ambiguous bases (e.g., N), as these indicate sequence uncertainty and may harbour unintended cut sites. To evaluate tool performance in such scenarios, we searched for the sgRNA GGAAGACACACTGGCAGAAANGG with $k = 0$ against a mock sequence where the sgRNA base at position 14 (C) was replaced by N in one version of a text, and by Y in another. Sassy implements the IUPAC profile for CRISPR off-target searches and returned all matches according to IUPAC base pairing. CHOPOFF requires a user to specify the maximum number of ambiguous bases (we used `-ambig-max=23`) and did also return all matches. SWOffinder does not have a command line option but a hardcoded boolean flag (default is `false`) which we set to `true` and recompiled. It did find the N version but not the Y version. Therefore, both Sassy and CHOPOFF have IUPAC support, and SWOffinder only supports N with source code modification. This result underscores the importance of selecting tools that correctly handle ambiguous bases in clinically relevant analyses.

E. Comparison with parasail

We compared Sassy to parasail, a SIMD-based affine-cost aligner [Daily, 2016], and to Edlib. To minimize overhead, we used the Rust bindings at `https://github.com/nsbuitrago/parasail-rs` to call parasail as a library, consistent with our setup for Sassy and Edlib.

Because affine scoring is not directly comparable to edit distance, we approximated edit distance costs by setting `-gap-open=1`, `-gap-extension=1`, `match-score=0`, and `mismatch-score=-1`. Given the small range of scores under this configuration, we used 8-bit output (`solution_width=8`).

parasail supports diagonal, striped, and prefix-scan vectorization. We used prefix-scan as it was the fastest for increasing pattern and text lengths.

As shown in Figure 8, when searching a text of $n = 100\,000$ bp with varying pattern lengths, Sassy achieves approximately $10\times$ higher throughput than Edlib and $100\times$ higher throughput than parasail. Similar trends hold when varying the text length (Figure 9).

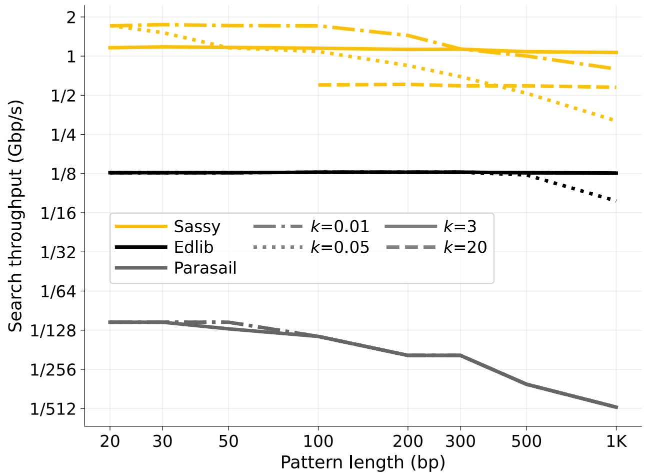

Fig. 8: Throughput of searching patterns of varying length. The pattern length m (x-axis) ranges from 20 to 1000, and the error threshold k (line style) is either fixed at 3 or 20, or computed as $\lceil m/100 \rceil$ or $\lceil m/20 \rceil$. Only points with $m > 3k$ are shown to avoid spurious matches. All points are computed by averaging over 1000 random patterns and texts of length $n = 10^5$, and then converting to throughput. Note that this does not include searching the reverse-complement strand. Sassy achieves up to $10\times$ higher throughput than Edlib for small k, and roughly two to three orders of magnitude higher throughput than parasail. The performance gap with parasail increases with pattern length, exceeding $500\times$ at $m = 1000$.

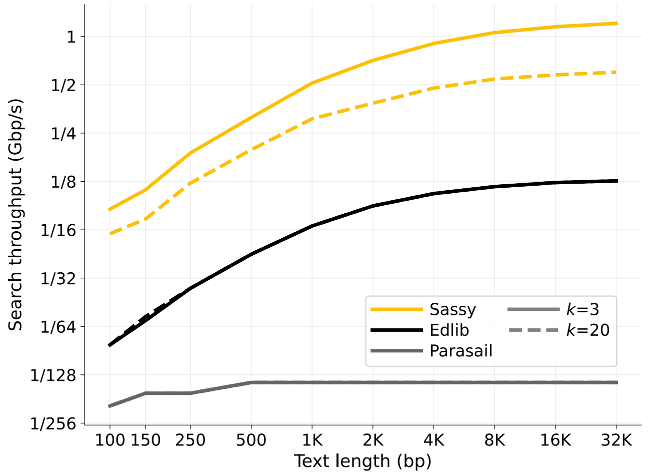

Fig. 9: Throughput of searching texts of varying length. We search a pattern of length $m = 100$ against texts with length varying from $n = 100$ to $n = 32\,000$ bp, with $k \in \{3, 20\}$. All points are computed by averaging over 1000 random texts and then converting to throughput. Note that this does not include searching the reverse-complement strand. Sassy consistently outperforms Edlib by about one order of magnitude, while parasail remains roughly two to three orders of magnitude slower but less sensitive to text length.
